# Role of glycogen synthase kinase 3 beta (GSK3β) in mediating the cytotoxic effects of the histone deacetylase inhibitor trichostatin A (TSA) in MCF-7 breast cancer cells

**DOI:** 10.1186/1476-4598-5-40

**Published:** 2006-10-03

**Authors:** John P Alao, Alexandra V Stavropoulou, Eric W-F Lam, R Charles Coombes

**Affiliations:** 1 Department of Cell and Molecular Biology, Lundberg Laboratory, Gothenburg University, P.O. Box 462, 405 30, Gothenburg, Sweden; 2 Department of Cancer Medicine, Cancer Cell Biology Section, Imperial College, Hammersmith Hospital, Du Cane Road, London, W12 0NN, UK

## Abstract

Histone deacetylase inhibitors (HDACIs) have been shown to induce apoptotic and autophagic cell death *in vitro *and *in vivo*. The molecular mechanisms that underlie these cytotoxic effects are not yet clearly understood. Recently, HDACIs were shown to induce Akt dephosphorylation by disrupting HDAC-protein phosphatase 1 (PP1) complexes. This disruption results in the increased association of PP1 with Akt, resulting in the dephosphorylation and consequent inactivation of the kinase. Akt enhances cellular survival through the phosphorylation-dependent inhibition of several pro-apoptotic proteins. Akt is an important negative regulator of GSK3β, a kinase that has been shown to regulate apoptosis in response to various stimuli. In the present study, we investigated the role of GSK3β in mediating the cytotoxic effects in MCF-7 breast cancer cells treated with trichostatin A (TSA), a prototype HDACI. We show that TSA induces Akt dephosphorylation in a PP1-dependent manner, resulting in activation of GSK3β in MCF-7 cells. Similarly, knockdown of HDAC1 and-2 by small interfering RNA (siRNA) resulted in the dephosphorylation of Akt and GSK3β. Selective inhibition of GSK3β attenuated TSA induced cytotoxicity and resulted in enhanced proliferation following drug removal. Our findings identify GSK3β as an important mediator of TSA-induced cytotoxicity in MCF-7 breast cancer cells.

## Findings

Histone deacetylase inhibitors (HDACIs) have been shown to induce apoptotic and autophagic cell death *in vitro *and *in vivo *[1-3]. The molecular mechanisms that underlie these cytotoxic effects are not yet clearly understood. Recently, HDACIs were shown to induce Akt (also called protein kinase B/PKB) dephosphorylation by disrupting HDAC-protein phosphatase 1 (PP1) complexes [4]. This disruption results in the increased association of PP1 with Akt, resulting in the dephosphorylation and consequent inactivation of the kinase. Akt enhances cellular survival through the phosphorylation-dependent inhibition of several proapoptotic proteins [5-7]. Mutation of negative regulators of Akt [8] and the deregulated expression or activation of Akt have been demonstrated in several cancers [9]. In addition, Akt activation has been shown to be associated with chemoresistance [10]. Phosphorylated, active Akt relocalizes to several cellular compartments where it phosphorylates a large number of substrates including FOXO transcription factors, GSK3, MDM2, BAD, TSC2, p70^S6K^, ASK1 p21^WAF1/Cip1^, p27^Kip1 ^and IKKα [6,10]. Akt is an important negative regulator of GSK3β, a kinase that has been shown to mediate apoptosis in response to various stimuli [11-15]. Akt phosphorylates GSK3β on Ser9 and inhibits its activity [16,17]. Recently, GSK3β was shown to be important for mediating the cell cycle effects of rapamycin and chemosensitivity to paclitaxel in MCF-7 cells [18]. We have previously demonstrated a role for GSK3β in mediating the effect of TSA on cyclin D1 levels in this cell line [19,20]. In the present study, we investigated the role of GSK3β in mediating cytotoxicity in MCF-7 breast cancer cells treated with trichostatin A (TSA), a prototype HDACI.

**Figure 1:**
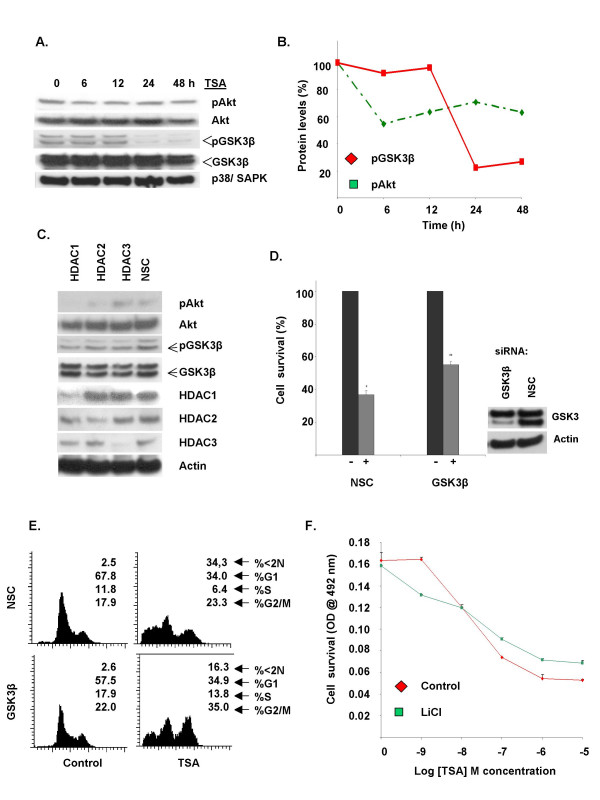
(A). TSA induces Akt and GSK3β dephosphorylation in MCF-7 breast cancer cells. MCF-7 cells were incubated with 1 μM TSA for the indicated times. Following incubation, the cells were harvested and lysates were resolved by SDS-PAGE. Proteins were detected using the indicated antibodies. (B). The relative amounts of pAkt and pGSK3β in A were measured by densitometry and normalised to the amount of p38/SAPK. Result is representative of at least three separate experiments. (C). Knockdown of class I HDAC proteins induces Akt and GSK3β dephosphorylation. MCF-7 cells were transfected with oligo pools specifically targeting HDAC1, 2, 3 or a non-targeting siRNA pool (NSC). 72 h after transfection, cells were harvested and lysed. Lysates were treated as in A and probed with the indicated antibodies. (D). siRNA-mediated GSK3β knockdown attenuates the cytotoxic effect of TSA on MCF-7 cells. MCF-7 cells were transfected with oligo pools specifically targeting GSK3β or a non-targeting siRNA pool (NSC). 24 h after transfection cells were harvested and reseeded in 96-well plates and incubated for 24 h. Cells were then treated with 1 μM TSA for 48 h and relative cell survival was measured as described in materials and methods. Results represent the mean ± S.E. from at least three separate experiments. * *P *< 0.001 TSA treated vs. untreated NSC siRNA cells, ** *P *< 0.001 TSA treated NSC vs. TSA treated GSK3β siRNA cells. Inset: Lysates from cells transfected in parallel were probed with antibodies directed against GSK3 to monitor siRNA efficiency. (E). Effect of GSK3β siRNA on TSA induced cytotoxicity. Cells were treated as in D and examined by flow cytometry (see materials and methods section). Result is representative of at least three separate experiments. (F). Effect of GSK3β inhibition on TSA induced cytotoxicity. MCF-7 cells were cultured in 96-well plates with 10^-9 ^– 10^-5 ^M TSA alone or in combination with 10 mM LiCl. Relative cell survival was determined after 48 h as described in materials and methods section. Result is representative of three separate experiments.

The treatment of U87MG glioblastoma and PC3 prostate cancer cells with HDAC inhibitors has been shown to induce the PP1-dependent dephosphorylation of Akt [4]. We investigated the effect of TSA on Akt and GSK3 phosphorylation in MCF-7 cells. Culture with 1 μM TSA for 24 h resulted in dephosphorylation of both kinases (Figure 1a). Similar experiments using selective inhibitors of c-Raf (ZM336372, 1 μM), p38 SAPK (SB203580, 10 μM), Erk1/2 (PD98059, 20 μM; U0126, 10 μM) and EGFR (genistein, 10 μM) did not result in GSK3β dephosphorylation (data not shown). In order to verify that Akt inhibition is sufficient to induce the loss of GSK3β dephosphorylation on Ser9, MCF-7 cells were treated with a specific Akt inhibitor. Culture of MCF-7 cells with 50 μM triciribine/TCN [21] reduced the levels of GSK3β phosphorylation on Ser9 (see additional file). To determine the role of phosphatases in mediating Akt and GSK3β dephosphorylation in MCF-7 cells, we investigated the effect of tautomycin and okadaic acid on the phosphorylation of these kinases. Tautomycin is specific for PP1 while low doses (≤ 5 nM) of okadaic acid selectively inhibit PP2A [22,23]. Culture of MCF-7 cells with tautomycin but not low dose okadaic acid resulted in increased phosphorylation levels of Akt and GSK3β. Co-culture of MCF-7 cells with TSA and tautomycin inhibited Akt and GSK3β dephosphorylation (see additional file 1). Taken together, our findings indicate that TSA induces GSK3β activation by mediating the PP1-dependent dephosphorylation of Akt in MCF-7 cells.

HDAC inhibitor induced disruption of PP1-HDAC complexes has been linked to protein kinase dephosphorylation [4]. We investigated the effect of class I HDAC knockdown by siRNA on protein kinase phosphorylation in MCF-7 cells. We used commercially available siRNA oligo pools specifically targeting HDACs 1, 2 and 3 as well as a non-targeting scrambled control oligo pools. Knockdown of HDAC1 and to a lesser extent HDAC2 but not HDAC3 resulted in Akt dephosphorylation. We observed that the knockdown of HDAC2 resulted in a partial reduction of HDAC1 levels and this may account for the effect of HDAC2 siRNA on Akt phosphorylation. Knockdown of HDAC1 and to a lesser extent HDAC2 and HDAC3 was sufficient to significantly reduce the phosphorylation levels of GSK3β (Figure 1c) compared with cells transfected with the non-targeting scrambled control oligos.

Akt facilitates cellular proliferation and survival by negatively regulating several proapoptotic molecules. GSK3β mediates apoptosis in response to various stimuli and is inhibited by Akt which phosphorylates the kinase on serine residue 9. The observation that HDAC inhibition leads to the dephosphorylation of Akt and GSK3β, suggested that GSK3β may mediate TSA-induced cytotoxicity in MCF-7 cells. Specific knockdown of GSK3β by siRNA significantly rescued MCF-7 from cell death following treatment with TSA (Figure 1d and 1e). FACS analyses demonstrated an increase in the S- and G2/M phase population following siRNA mediated GSK3β knockdown in untreated cells. GSK3β knockdown also resulted in a significant decrease in the sub-G1 cell population (~50%) compared to cells transfected with a non targeting oligo pool, following treatment with 1 μM TSA for 48 h. GSK3β knockdown appeared to attenuate the cytotoxic effects of TSA on cells in the S- and G2/M cell cycle phases [24] resulting in the increased survival of these populations (13.8% and 35.0% in S- and G2/M respectively for GSK3β siRNA transfected cells vs. 6.4% and 23.3% in the control siRNA transfected population) (Figure 1e). Co-treatment of MCF-7 cells with TSA and the GSK3β specific inhibitors SB216763 and lithium chloride (LiCl) also resulted in enhanced survival compared to cells treated with TSA alone (Figure 1f and additional file 1). As expected, SB216763 and LiCl also inhibited tricribine/TCN induced cytotoxicity (data not shown). Interestingly, LiCl enhanced the antiproliferative effect of TSA at sub-cytotoxic concentrations (<10 nm) but significantly enhanced survival at concentrations above 10 nM (Figure 1f). These observations demonstrate that GSK3β is an important mediator of TSA induced apoptosis in MCF-7 cells and that inhibition of its activity significantly enhances survival of these cells following exposure to TSA.

We have shown that TSA induces Akt dephosphorylation in a PP1-dependent manner, resulting in activation of GSK3β in MCF-7 cells. Similarly, knockdown of HDAC1 and 2 by small interfering RNA (siRNA) resulted in the dephosphorylation of Akt and GSK3β. Selective inhibition of GSK3β attenuated TSA induced cytotoxicity. HDAC inhibitors have proved promising as anti-cancer agents in both *in vitro *and *in vivo *studies. The precise mechanisms that underlie their cytostatic and cytotoxic activities remain poorly defined. Understanding these mechanisms is however important for the design of more specific HDAC inhibitors. In addition, a better understanding of the molecular pharmacology of these inhibitors will aid in the identification of those cancer subtypes where their application is likely to be most effective from a clinical standpoint. While GSK3β has been shown to mediate apoptosis in several cell types, its role in mediating cytotoxicity in MCF-7 breast cancer cells has only been recently demonstrated [18]. In that study, GSK3β was shown to be important for mediating rapamycin-dependent chemosensitization. Furthermore, compounds that specifically inhibit GSK3β (SB216763, SB415286) were found to interfere with rapamycin-mediated paclitaxel sensitization or cell cycle arrest (LiCl). Our findings identify GSK3β as an important mediator of TSA-induced apopotosis in MCF-7 breast cancer cells. Inhibition of GSK3β with the selective inhibitor SB216763 (as well as LiCl) significantly inhibited the cytotoxic effect of TSA on this cell line. While the use of GSK3β specific inhibitors has not been linked to the development of cancer, our observations provide further evidence for the potential of these compounds to interact negatively with anti-cancer therapeutics.

## Materials and methods

### Reagents

Stock solutions of TSA (Sigma-Aldrich; Dorset, United Kingdom) in ethanol were stored at -20°C. The GSK3-specific inhibitor SB216763 (Tocris Bioscience, Avonmouth, United Kingdom) was dissolved in DMSO and stored at -20°C. ZM336372, PD98059, SB203580, U0126 and genistein were purchased as 10 mM stock solutions dissolved in DMSO and stored at -20°C (Tocris bioscience). Lithium Chloride (Sigma-Aldrich) was dissolved in sterile distilled water and stored at 4°C. The phosphatase inhibitors okadaic acid and tautomycin (Calbiochem, Beeston, Nottingham, United Kingdom) were dissolved in DMSO and stored at -20°C. Antibodies to actin (Santa Cruz Biotechnology, Santa Cruz, CA), phosphor-Akt, Akt, phosphoGSK3β, GSK3β (Upstate Biotechnology, Dundee, United Kingdom), p38/SAPK (New England Biolabs, Hitchin, United Kingdom), and HDAC1, HDAC2, HDAC3 (Abcam, Cambridge, United Kingdom) were used.

### Cell culture and treatments

MCF-7 cells (American Type Culture Collection, Rockville, MD) were cultured in DMEM supplemented with 10% (v/v) fetal calf serum, 2 mM L-glutamine, 100 units/ml penicillin and 100 μg/ml streptomycin at 37°C in humidified 5% CO_2_.

### Cell proliferation assay

Cells were seeded in 96-well plates at a predetermined optimal cell density to ensure exponential growth for duration of the assay. After a 24 h preincubation, growth medium was replaced with experimental medium containing the appropriate drug concentrations or 0.1% (v/v) vehicle control. After a 48 h incubation, cell proliferation was estimated using the sulforhodamine B colorimetric assay [25] and expressed as the mean ± SD for six replicates as a percentage of vehicle control (taken as 100%). Experiments were performed independently at least three times. Statistical analyses were performed using a two-tailed Student's *t *test. *P *< 0.05 was considered to be statistically significant.

### Immunoblotting

Cells treated as indicated were harvested in 5 ml of medium, pelleted by centrifugation (1,000 × *g *for 5 min at 4°C), washed twice with ice-cold PBS and lysed in ice-cold HEPES buffer [50 mM HEPES (pH 7.5), 10 mM NaCl, 5 mM MgCl_2_, 1 mM EDTA, 10% (v/v) glycerol, 1% (v/v) Triton X-100 and a cocktail of protease inhibitors] on ice for 30 min. Lysates were clarified by centrifugation (15,000 × *g *for 10 min at 4°C) and the supernatants then either analyzed immediately or stored at -80°C. Equivalent amounts of protein (20–50 μg) from total cell lysates were resolved by SDS-PAGE using precast 4–12% Bis-Tris gradient gels (Invitrogen Ltd., Paisley, United Kingdom) and transferred onto polyvinylidene difluoride (PVDF) membranes (Hybond P; Amersham Biosciences United Kingdom Limited, Little Chalfont, United Kingdom) with a Novex XCell system (Invitrogen). Membranes were blocked overnight at 4°C in blocking buffer [5% (w/v) nonfat dried milk, 150 mM NaCl, 10 mM Tris (pH 8.0) and 0.05% (v/v) Tween 20]. Proteins were detected by incubation with primary antibodies at appropriate dilutions in blocking buffer overnight at 4°C. Blots were then incubated at room temperature with horseradish peroxidase-conjugated secondary antibody. Bands were visualized by enhanced chemiluminescence (Supersignal West Pico; Perbio Science UK Ltd., Cheshire, United Kingdom) followed by exposure to autoradiography film (Kodak BioMax ML-light or MR-1). The relative amounts of protein levels were measured densitometrically using Image Quant™ software (GE Healthcare UK Ltd., Little Chalford, United Kingdom) and normalised to the level of p38/SAPK (loading control).

### Flow cytometry

MCF-7 cells were treated as indicated. Floating and adherent cells were collected by centrifugation (500 × g for 5 minutes at 4°C) and washed twice with PBS. Cells were fixed in 90% ethanol and stored at -20°C. For analysis, cells were washed in PBS and stained by resuspension in propidium iodide (PI, 50 μg/mL) in water containing RNase A (2 μg/mL) for 30 min at 4°C. Single cell suspensions were analysed on a FACScantor cytometer (BD Biosciences Immunocytometry Systems, San Jose, CA). with CellQuest (BD Biosciences) acquisition software. PI fluorescence was measured through a 585/42 nm band pass filter, and list mode data were acquired on a minimum of 10,000 single cells defined by a dot blot of PI width *versus *PI area.

### siRNA transfection

MCF-7 cells were transfected with commercially available siRNA oligonucleotide pools (Dharmacon, Lafayette, CO) using Oligofectamine transfection reagent (Invitrogen, Groningen, The Netherlands) as previously described [26]. Fugene 6 transfection reagent (Roche Diagnostics Ltd, East Sussex, United Kingdom) was used for DNA plasmid transfection. Asynchronous cell populations at a density of 50–60% in 6-well plates or on coverslips were transfected with 1–2 μg of plasmid DNA, following the formation of lipid-DNA complexes for 20 min at room temperature in Optimem I medium (Invitrogen). Complexes were added directly to cells growing in 2 ml DMEM and incubated for 5 h followed by washing with PBS buffer and addition of fresh DMEM. Cells were normally used in experiments 24 h following transfection and the recombinant proteins detected by immunoblotting.

## Abbreviations

DMEM-Dulbecco's modified eagle medium, DMSO-dimethyl sulphoxide, Erk-extracellular regulated kinase, FACS-fluorescent activated cell sorting, GSK3β-glycogen synthase 3 beta, HDAC-histone deacetylase, OA-okadaic acid, PBS-phosphate buffered saline, PI-propidium iodide, PKB-protein kinase B, siRNA-small inhibitory RNA, SAPK-stress activated protein kinase, TCN-triciribine, TM-tautomycin, TSA-trichostatin A.

## Competing interests

The author(s) declare that they have no competing interests.

## Authors' contributions

JPA, DMV, EW-FL and RCC conceived of the study, coordinated its design and execution and drafted the manuscript. JPA and AVS carried out survival assays, siRNA, immunoblot experiments and FACS. JPA, AVS and EW-FL interpreted and analyzed the data. All authors read and approved the final draft manuscript.

## Supplementary Material

Additional file 1 GSK3β mediates TSA-induced cytotoxicity in MCF-7 breast cancer cells. Additional file 1 (A). Specific inhibition of Akt is sufficient to induce GSK3β dephosphorylation in MCF-7 breast cancer cells. MCF-7 cells were incubated with 1 μM TSA or 50 μM triciribine (TCN) for 24 h. Following incubation, the cells were harvested and lysates were resolved by SDS-PAGE. Proteins were detected using the indicated antibodies. (B) Specific inhibition of protein phosphatase 1 (PP1) enhances Akt and GSK3β phosphorylation. MCF-7 cells were incubated for 24 h with 5 μM tautomycin, 10 nm okadaic (OA 10) or 100 nm okadaic acid (OA 100). Following incubation, the cells were harvested and lysates were resolved by SDS-PAGE. Proteins were detected using the indicated antibodies. (C) Tautomycin inhibits TSA induced Akt and GSK3β dephosphorylation. MCF-7 cells were treated with 1 μM TSA alone or in combination with 5 μM tautomycin for 24 h. Proteins were detected using the indicated antibodies. (E, F) Specific inhibition of GSK3β attenuates TSA-induced cytotoxicity in MCF-7 cells. Cells were treated for 48 h with 1 μM TSA alone and in combination with the GSK3β inhibitor SB216763 (5 and 10 μM) (SB5, SB10) or 10 mM LiCl. Relative cell survival was measured as described in material and methods section. Results represent the mean ± S. E. from three separate experiments. *P < 0.05, *P *< 0.01, TSA treated vs. TSA with 5 and 10 μM SB216763 treated cells respectively, *P *< 0.0001, TSA treated vs. TSA and LiCl treated cells. Click here for file
