# Supplementary material for: Role of glycogen synthase kinase 3 beta (GSK3β) in mediating the cytotoxic effects of the histone deacetylase inhibitor trichostatin A (TSA) in MCF-7 breast cancer cells
Source: Mol Cancer. 2006 Oct 3;5:40. doi: 10.1186/1476-4598-5-40 (PMC1592505; doi:10.1186/1476-4598-5-40)
Supplement: Additional file 1 — GSK3β mediates TSA-induced cytotoxicity in MCF-7 breast cancer cells. Additional file 1 (A). Specific inhibition of Akt is sufficient to induce GSK3β dephosphorylation in MCF-7 breast cancer cells. MCF-7 cells were incubated with 1 μM TSA or 50 μM triciribine (TCN) for 24 h. Following incubation, the cells were harvested and lysates were resolved by SDS-PAGE. Proteins were detected using the indicated antibodies. (B) Specific inhibition of protein phosphatase 1 (PP1) enhances Akt and GSK3β phosphorylation. MCF-7 cells were incubated for 24 h with 5 μM tautomycin, 10 nm okadaic (OA 10) or 100 nm okadaic acid (OA 100). Following incubation, the cells were harvested and lysates were resolved by SDS-PAGE. Proteins were detected using the indicated antibodies. (C) Tautomycin inhibits TSA induced Akt and GSK3β dephosphorylation. MCF-7 cells were treated with 1 μM TSA alone or in combination with 5 μM tautomycin for 24 h. Proteins were detected using the indicated antibodies. (E, F) Specific inhibition of GSK3β attenuates TSA-induced cytotoxicity in MCF-7 cells. Cells were treated for 48 h with 1 μM TSA alone and in combination with the GSK3β inhibitor SB216763 (5 and 10 μM) (SB5, SB10) or 10 mM LiCl. Relative cell survival was measured as described in material and methods section. Results represent the mean ± S. E. from three separate experiments. *P < 0.05, P < 0.01, TSA treated vs. TSA with 5 and 10 μM SB216763 treated cells respectively, P < 0.0001, TSA treated vs. TSA and LiCl treated cells. [file 1476-4598-5-40-S1.ppt]

## Slide 1
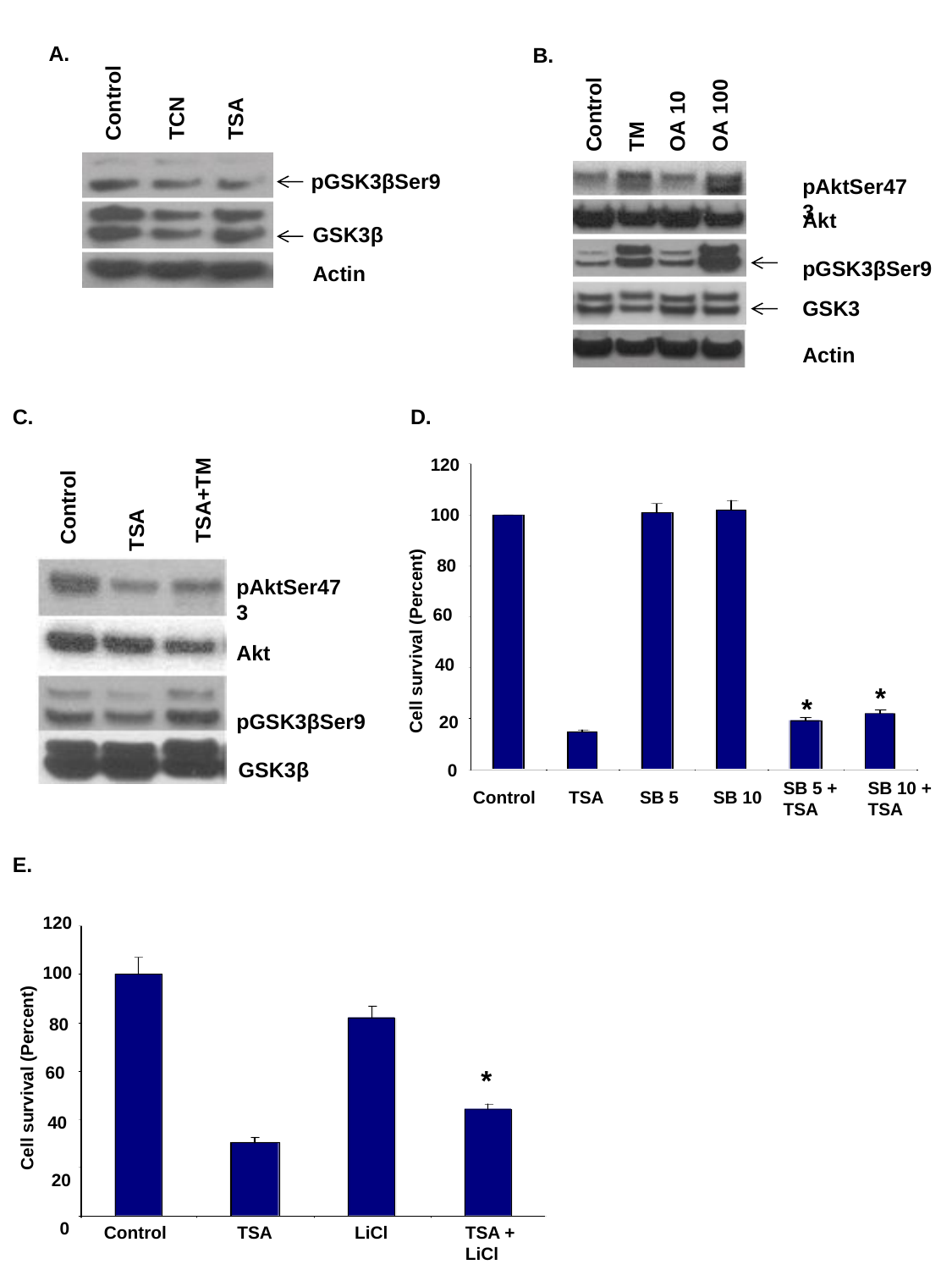

A.
B.
Control
OA 100
TSA
Control
TCN
OA 10
TM
pGSK3βSer9
pAktSer473
Akt
GSK3β
pGSK3βSer9
Actin
GSK3
Actin
C.
D.
TSA+TM
Control
TSA
pAktSer473
Akt
pGSK3βSer9
GSK3β
120
100
80
60
40
20
0
Cell survival (Percent)
 *
*
Control
TSA
SB 5
SB 10
SB 5 +
TSA
SB 10 +
TSA
E.
120
100
80
60
40
20
0
Cell survival (Percent)
*
Control
TSA
LiCl
TSA + LiCl
